# Neuropeptide Y and Calcitonin Gene-Related Peptide in Cerebrospinal Fluid in Parkinson’s Disease with Comorbid Depression versus Patients with Major Depressive Disorder

**DOI:** 10.3389/fpsyt.2017.00102

**Published:** 2017-06-12

**Authors:** Per Svenningsson, Sven Pålhagen, Aleksander A. Mathé

**Affiliations:** ^1^Department of Clinical Neuroscience, Karolinska Institutet, Stockholm, Sweden

**Keywords:** Parkinson’s disease, depression, non-motor symptoms, neuropeptides, biomarker

## Abstract

Parkinson’s disease (PD) is the second most common neurodegenerative disease in the world. The diagnosis of PD is based on movement dysfunctions. Many patients also suffer from comorbid depression in spite of adequate treatment with dopamine replacement, indicating that also other non-dopaminergic mechanisms are involved. Indeed, neuropeptides are critically implicated in the pathophysiology of major depressive disorder (MDD). To increase our understanding of the biochemical basis of depression in PD patients, we examined the levels of neuropeptide Y (NPY) and calcitonin gene-related peptide (CGRP) in cerebrospinal fluid (CSF) from PD patients, with or without comorbid depression, and compared them to the levels in patients with MDD. We also compared the levels of NPY and CGRP with 5-hydroxyindoleacetic acid (5-HIAA), the major serotonin metabolite. Both NPY and CGRP were higher in PD patients with comorbid depression compared to MDD patients. No similar difference was found in 5-HIAA levels. Accordingly, there were no correlations between NPY and 5-HIAA or CGRP and 5-HIAA levels. The finding of higher NPY and CGRP CSF levels in PD patients with MDD raises the possibility that different pathophysiological processes may underlie depression in PD and MDD.

## Introduction

Parkinson’s disease (PD) is a debilitating inexorably progressing neurodegenerative disorder affecting about 1% of persons above the age of 65 (1). PD is characterized by a progressive loss of nigrostriatal dopaminergic neurons in the substantia nigra pars compacta resulting in bradykinesia, rigidity, and resting tremor (1). In addition to the motor symptoms, PD patients also suffer from non-motor symptoms, including depression, hyposmia, sleep disorders, autonomic dysfunctions, hallucinations, and cognitive impairments (2). Depression is common at all stages of PD, ranging between 10 and 70%, the prevalence rate depending on the criteria used to define depression (3). There is a poor understanding of the pathophysiology underlying depression in PD, resulting in a lack of consensus on the therapeutic antidepressant strategies (3). There are no separate specific guidelines for treatment of major depressive disorder (MDD) in PD and patients have been treated according to the best practice for treatment of MDD patients without PD. Hypofunctional dopamine neurotransmission does not only cause movement dysfunctions in PD but also anhedonia and loss of motivation, which characterizes depression in PD (2). However, depression is also seen in PD patients adequately treated with dopamine replacement, indicating that non-dopaminergic mechanisms are involved. It has been difficult to establish whether serotonin transmission plays a critical role in depression in PD. Positron emission tomography imaging studies have shown that the availability of the serotonin transporter was higher in the raphe nuclei and limbic regions of patients with PD who had depression compared with non-depressed PD patients (4, 5), although this finding was not evident in all studies (6). Likewise, studies of the serotonin metabolite 5-hydroxyindoleacetic acid (5-HIAA) in cerebrospinal fluid (CSF) from PD patients, with or without depression, have yielded contradictory results. Some studies have found reduced levels (7, 8), whereas others found no association between the 5-HIAA CSF levels and the presence and/or severity of depression in patients with PD (9, 10). Research in MDD has provided a wealth of evidence that, in addition to monoamine dysregulation, other compounds such as stress hormones, immune mediators, neurotrophic factors, and neuropeptides underlie different aspects of the depressive symptomatology (11).

Neuropeptide Y (NPY), an evolutionary well preserved 36 amino acid peptide, abundantly present in the brain, modulates a wide variety of brain functions under physiological conditions and is altered in several affective brain disorders (12, 13). Ample evidences show that NPY is reduced in postmortem brain as well as CSF from subjects who suffered from unipolar or bipolar depressive disorder (14–18).

Another neuropeptide of relevance is the 37 amino acid calcitonin gene-related peptide (CGRP), widely distributed in the brain. Alternative splicing of the initial calcitonin gene transcripts results in production of two different mRNAs encoding CGRP or calcitonin/katacalcin (19), with CGRP being the predominant transcript in neural tissues (20). There is a paucity of data regarding CGRP in human brain and CSF; however, elevated CGRP concentrations were found in unipolar and bipolar depressed patients and in patients diagnosed with Alzheimer’s disease (21, 22). Several studies have demonstrated extensive interactions between CGRP and dopamine neurotransmission. When administered directly into rat CNS, CGRP markedly affects dopamine release and metabolism in selected brain regions (23, 24). High concentrations of CGRP are measured in neuronal cells in, e.g., ventral tegmental area, while CGRP fibers are found most abundantly in dopaminoceptive areas (20). The receptors, CGRP-1 and CGRP-2, are selectively distributed in the CNS, often localized on the dopaminergic neurons (25, 26).

Taken together, these findings indicate a potential role for CGRP in dopamine-related CNS disorders, such as PD. In view of the above findings and since NPY and CGRP have apparently not been explored in PD, we examined their levels in CSF from PD patients, with or without comorbid depression, and compared them to the levels in patients with MDD. We also correlated the CSF levels of these neuropeptides with those of 5-HIAA.

## Materials and Methods

All work was approved by the Regional Review Board and in line with the rules and regulations of the Swedish law code and the Helsinki Declaration. As shown in Table 1, non-demented PD patients with (*n* = 10) or without (*n* = 12) comorbid depression and nine patients with MDD were studied. The structured clinical interview for DSM-III-R was used to identify depression. The severity of the depression was scored on the Montgomery–Åsberg Depression Rating Scale (MADRS). For depressed patients with or without PD, no antidepressive treatment was given 6 months prior to the study.

**Table 1:** Demographics of the studies patients.

Group	PD	PD + Dep	Dep
Sex (women/men)	4/8	7/3	5/4
Age (years)	65.3 ± 7.8	63.9 ± 10.4	64.2 ± 9.8
PD duration (years)	6.9 ± 2.5	9.6 ± 4.9	n.a.
MADRS	n.a.	16.6 ± 3.8	21.6 ± 5.5

*Values given as mean ± SD*.

*Dep, depression; MADRS, Montgomery–Åsberg Depression Rating Scale; n.a., not applicable; PD, Parkinson’s disease*.

### Analysis of CSF Samples

A standardized lumbar puncture procedure was performed at the L4–5 level with the patient in a supine position. Between 12 and 15 mL CSF from the first portion was collected to minimize the gradient influence. Samples were collected between 1998 and 2001, and aliquots were stored in −80°C freezer until assayed in 2009 (for 5-HIAA) and in 2011 (for NPY and CGRP). NPY and CGRP were measured by radioimmunoassays as previously reported (17, 18, 21, 22). The level of 5-HIAA was determined as previously described (27) by a mass fragmentographic method using the deuterated metabolites as internal standards. To adjust for body height and age, the level of 5-HIAA was corrected with the following formulae: 5-HIAAcorr = 5-HIAA/[514 + (0.79 × age) − (2.5 × length)] × 100 (27).

### Data and Statistical Analysis

All data showed passed for normality and Gaussian distribution by the Kolmogorov–Smirnov test. Data are presented as mean ± SD. Data were analyzed with one-way ANOVAs followed by Newman–Keuls test for pairwise comparisons or Pearson correlation analyses (GraphPad Prism).

## Results

**Figure 1:**
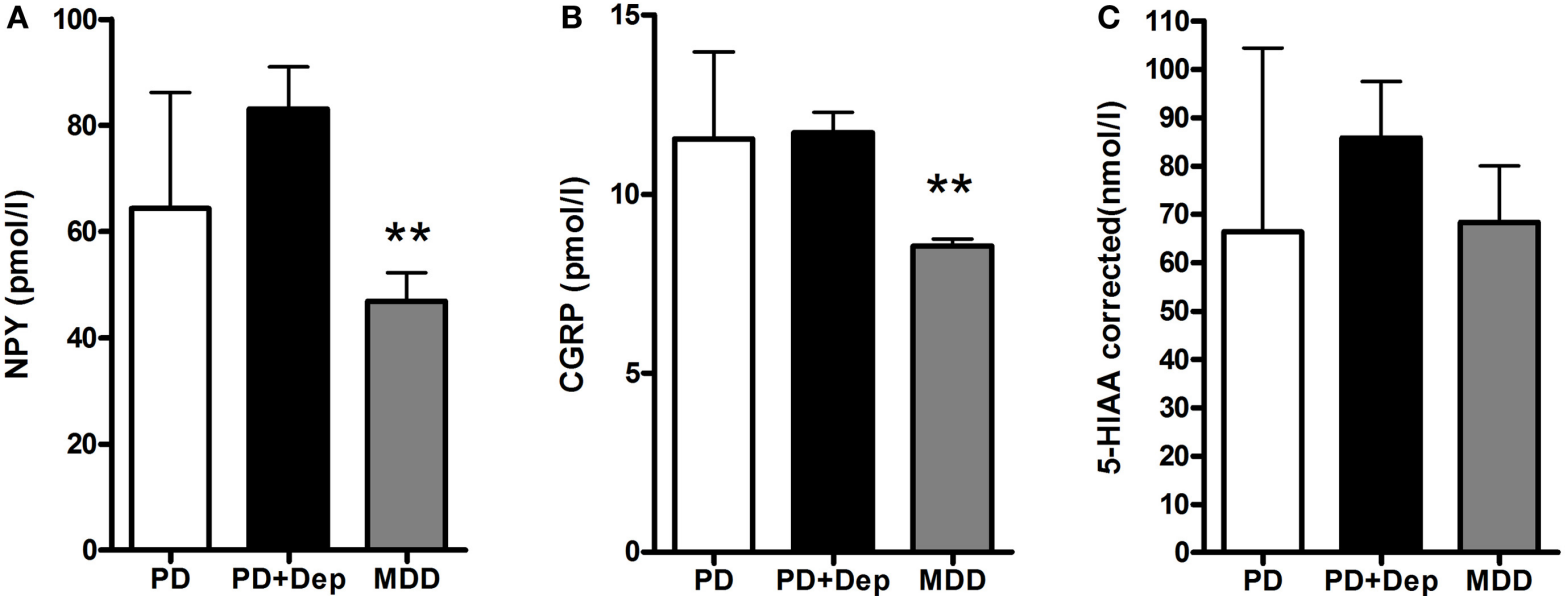
Levels of NPY, CGRP, and 5-HIAA in CSF in PD patients with and without depression and patients with MDD. There are lowered levels of NPY **(A)** and CGRP **(B)**, but not 5-HIAA **(C)** in the MDD patients. Data are presented as mean ± SD and analyzed using a one-way-ANOVA followed by Newman–Keuls test for pairwise comparisons. **P < 0.01 versus PD + Dep patients. Abbreviations: CGRP, calcitonin gene-related peptide; CSF, cerebrospinal fluid; Dep, depression; 5-HIAA, 5-hydroxyindoleacetic acid; MDD, major depressive disorder; NPY, neuropeptide Y; PD, Parkinson’s disease.

**Figure 2:**
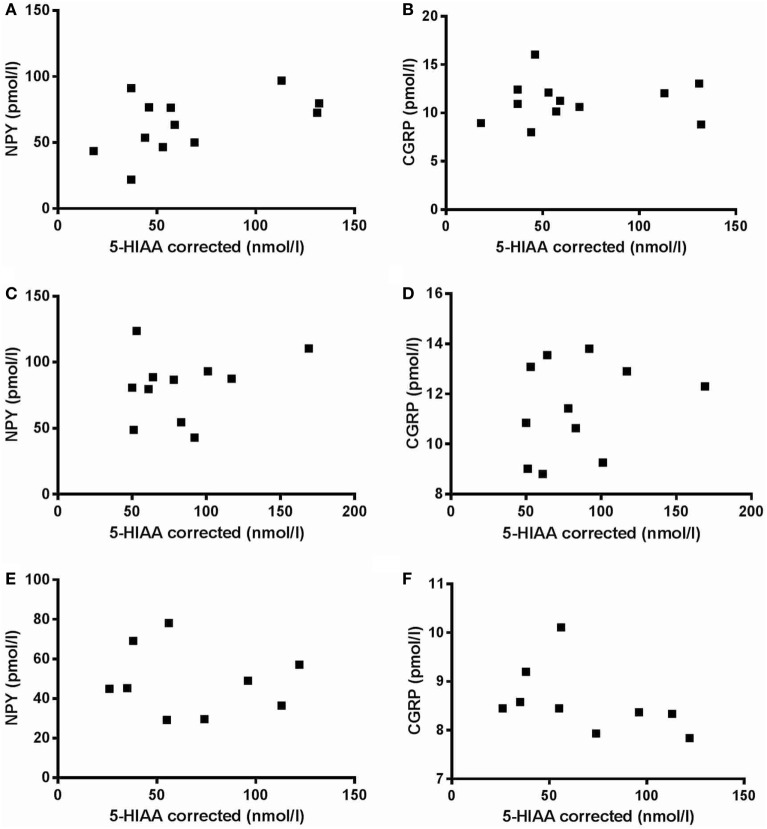
Correlation between neuropeptide Y (NPY) versus 5-hydroxyindoleacetic acid (5-HIAA) and calcitonin gene-related peptide (CGRP) versus 5-HIAA. There are no significant correlations between NPY versus 5-HIAA **(A,C,E)** or between CGRP versus 5-HIAA **(B,D,F)** in neither Parkinson’s disease patients without **(A,B)** or with **(C,D)** comorbid depression nor in major depressive disorder patients **(E,F)**.

There was no significant difference in severity of depression according to MADRS between PD patients with comorbid depression (16.6 ± 3.8) and MDD patients (21.6 ± 5.5). The levels of NPY in PD patients, without or with comorbid depression, and in patients with MDD were 64.4 ± 21.9, 83.1 ± 25.2, and 46.8 ± 17.0 pmol/L, respectively (Figure 1A). These levels are comparable to previous reports (17). One-way ANOVA revealed that the NPY levels differed between the groups (*F*_2,31_ = 6.99; *P* = 0.003), and *post hoc* analysis demonstrated significantly (*P* < 0.01) higher NPY levels in depressed PD patients compared to MDD patients. The levels of CGRP in PD patients, without or with comorbid depression, and in patients with MDD were 11.6 ± 2.4, 11.7 ± 1.9, and 8.5 ± 0.7 pmol/L, respectively (Figure 1B). These levels are comparable to previous reports (22). In similarity to the NPY data, one-way ANOVA revealed that the CGRP levels differed between the groups (*F*_2,32_ = 9.34; *P* = 0.001), and *post hoc* analysis demonstrated significantly (*P* < 0.01) higher levels of CGRP in depressed PD patients compared to MDD patients. The levels of 5-HIAA in PD patients, without or with comorbid depression, and in patients with MDD were 66.3 ± 38.1, 85.8 ± 36.9, and 68.3 ± 35.0 nmol/L, respectively (Figure 1C). One-way ANOVA revealed no statistical differences between the groups (*F*_2,28_ = 0.87; *P* = 0.43). These data have previously been reported (10). To study neuropeptide–serotonin interactions, we correlated the levels NPY or CGRP with those of 5-HIAA (Figures 2A–F). In PD patients, the correlations between NPY and 5-HIAA and CGRP and 5-HIAA were *r* = 0.50 and *r* = 0.06; in PD patients with comorbid depression, they were *r* = 0.25 and *r* = 0.26 and in MDD patients, they were *r* = −0.14 and *r* = −0.47. None of these correlations reached statistical significance.

## Discussion

These are the first data regarding the regulation of NPY and CGRP in PD and comparing these two peptides in PD with comorbid depression to MDD. Our results show that both NPY and CGRP in CSF are higher in PD patients with comorbid depression compared to MDD patients. Cumulative results do not consistently support 5-HIAA concentration decrease in MDD. High and low concentrations (that is a bimodal distribution) were originally reported (28). Subsequent studies found correlations to suicide as well as aggression and violent behavior but no clear correlation to depression (29). Consequently, the findings that NPY and CGRP did not correlate to 5-HIAA are not at variance with the data in literature and are in line with shifting the focus to dysregulation of other compounds and systems in MDD. Thus, a plethora of data shows that neuropeptides, the glutamatergic signaling, BDNF, changes in metabolome (e.g., leptin and insulin), neurogenesis, and glia pathology play significant roles. A comprehensive elucidation of depression neurobiology has not been accomplished, possibly due to the fact that clinical depression is a phenotype reflecting a number of underlying pathologies.

Notably, preclinical data consistently show reduced NPY in genetic and epigenetic models of depression, posttraumatic stress disorder (PTSD), and chronic stress, translationally confirmed in patients diagnosed with MDD and PTSD [cf. reviews (12, 13)]. In contrast to these findings, NPY was elevated in depressed PD patients. Extensive evidence points to interrelationships between NPY and the dopaminergic system; e.g., icv administered NPY increases dopamine concentrations in several brain regions and its release from striatum (30, 31). Conversely, acute activation of the dopaminergic system by administration of d-amphetamine increased basal extracellular NPY efflux in the ventral striatum, an effect that could be blocked by dopamine receptor antagonists (32). The mechanisms accounting for our finding of increased NPY have not been elucidated, but conceivable explanations are that treatment with l-DOPA affected the dopamine–NPY interrelationship and/or that the depression in PD is the end result of a different pathophysiology.

As reviewed in the Section “Introduction,” increased CSF levels of CGRP were found in depression and in patients diagnosed with Alzheimer’s (21, 22). Translationally, these results are in agreement with the increased CGRP in frontal cortex, amygdala, and hippocampus in Flinders sensitive line rats, a model of depression (33). Moreover, in microdialysis experiments acute administration of amphetamine or phencyclidine increased CGRP efflux (25, 26). Further, in two mouse strains, CGRP-injected ICV decreased depression-like behaviors in both strains (34). These findings suggest that CGRP may have an antidepressant action and it is possible that an elevated expression of CGRP reflects an adaptive response to the disease. Alternatively, since all PD patients were treated with levodopa and there is bidirectional regulation of dopamine and CGRP, levodopa therapy may have influenced levels of CGRP.

The limitation of the study is that we did not have a control group of matched healthy subjects and consequently can only compare CSF levels of NPY and CGRP to those found in MDD. The strength of the study is that, to the best of our knowledge, this is the first time that differences in NPY and CGRP CSF levels between PD patients with comorbid depression and MDD patients have been reported. Our results indicate that the pathophysiological mechanisms underlying depression in PD and MDD may not be identical and warrant further studies in animal models of PD as well as PD patients, in particular untreated *de novo* PD patients.

## Ethics Statement

All participants gave their written informed consent before entering the study. The study was approved and accepted by the regional ethics committee (Faculty of Health Sciences, Linköping University, Dnr 96165). All work was approved by the Regional Review Board and in line with the rules and regulations of the Swedish law code and the Helsinki Declaration.

## Author Contributions

PS and AM designed the study and have written up the manuscript. AM performed the analyses of NPY and CGRP. PS conducted the statistical analyses. SP saw the patients, obtained informed consent, and collected the CSF.

## Conflict of Interest Statement

The authors declare that the research was conducted in the absence of any commercial or financial relationships that could be construed as a potential conflict of interest.
